# The association between weekend catch-up sleep and prevalence of chest pain in U.S. adults: A cross-sectional analysis of NHANES 2017 to 2020

**DOI:** 10.1097/MD.0000000000045785

**Published:** 2025-11-07

**Authors:** Chong Zhao, Jinjin Jian, Jing Zheng, Qi Wang, Jinfang Zeng

**Affiliations:** a Department of Anesthesiology, Sir Run Run Shaw Hospital, Zhejiang University School of Medicine, Hangzhou, China; b Department of Anesthesiology, Affiliated Hospital of Jiangnan University, Wuxi, China; c Department of Thyroid and Breast Surgery, Affiliated Hospital of Jiangnan University, Wuxi, China; d Department of Radiation Oncology, Affiliated Hospital of Jiangnan University, Wuxi, China; e Department of Anesthesiology and Pain Medicine, Jiangnan University Medical Center (Wuxi No. 2 People’s Hospital, Affiliated Wuxi Clinical College of Nantong University), Wuxi, China.

**Keywords:** cardiovascular health, chest pain, NHANES, sleep deprivation, weekend catch-up sleep

## Abstract

Weekend catch-up sleep (WCS) – extending sleep on nonworking days to compensate for weekday deficits – has been linked to cardiometabolic health. However, its association with chest pain, a common and clinically significant symptom, remains unclear. Data from the 2017 to 2020 National Health and Nutrition Examination Survey were used to assess this relationship. We analyzed 6330 U.S. adults, categorizing WCS (self-reported weekend-weekday sleep difference) into quartiles: Q1 (<0 hours), Q2 (0–1 hour), Q3 (1–2 hours), and Q4 (≥2 hours). Chest pain was assessed through validated questionnaires. The association was evaluated using weighted multivariable logistic regression, restricted cubic splines, and subgroup analyses, accounting for demographics, socioeconomic background, lifestyle, and clinical conditions. A dose–response relationship was observed, with longer WCS linked to lower chest-pain prevalence. Compared to Q1, Q4 participants had 29% lower odds of chest pain (Odds Ratio (OR) = 0.71, 95% confidence interval (CI): 0.58–0.87, *P* = .001). Each additional hour of WCS reduced the risk by 8% (OR = 0.92, 95% CI: 0.88–0.96, *P* <.0001). Subgroup analyses revealed stronger associations in hypertensive individuals (OR = 0.88, 95% CI: 0.83–0.93, *P* <.001) and those married or cohabiting (OR = 0.87, 95% CI: 0.82–0.93, *P* <.001). Nonlinear modeling confirmed an inverse trend without a clear threshold (*P* for nonlinearity = 0.076). This cross-sectional study suggests that WCS is independently associated with reduced chest pain, particularly in hypertensive and partnered individuals. While these findings indicate potential cardiovascular relevance of compensatory sleep, further prospective research is warranted to clarify the nature of this association and its clinical implications. Interventions aimed at promoting adequate sleep recovery may be considered for reducing chest-pain risk in vulnerable populations, pending confirmation from longitudinal studies.

## 1. Introduction

Chest pain is a major cause of emergency department visits in the United States, affecting over 8 million individuals annually and accounting for 5% to 12% of all visits.^[1]^ Beyond its acute clinical urgency, chest pain serves as a prognostic marker for cardiovascular outcomes, with a lifetime prevalence of 20% to 40%.^[2,3]^ Despite its importance, risk stratification remains difficult due to its heterogeneous etiologies, highlighting the need to identify modifiable risk factors.

Sleep has emerged as a modifiable factor in cardiovascular health. Both short and long sleep durations are associated with increased risks of adverse outcomes, including chest pain, heart failure, and stroke.^[4,5]^ Our findings suggest that sleep may serve as an important factor in cardiovascular risk assessment.

Sleep irregularity, such as weekday–weekend discrepancies (“sleep debt”), has been linked to poorer cardiovascular health metrics.^[6]^ In addition, poor sleep quality has been associated with cardiometabolic symptoms that may overlap with or worsen chest discomfort.^[7]^

Reflecting these associations, the American Heart Association now recognizes sleep as an essential component of cardiovascular health.^[8]^ Within this framework, weekend catch-up sleep (WCS) has gained attention as a potential compensatory behavior for weekday sleep loss, though its cardiovascular effects remain uncertain.^[9,10]^

WCS, defined as extending sleep duration on nonworking days to compensate for weekday sleep debt, is a common behavior.^[11]^ Some studies suggest it may improve blood pressure, insulin sensitivity, and inflammatory profiles.^[12,13]^

However, recent prospective studies suggest that WCS may not reduce cardiovascular disease incidence, and greater sleep discrepancies may even be linked to poorer cardiovascular metrics.^[6,11]^

In summary, while WCS may offer short-term physiological benefits, its long-term cardiovascular impact remains unclear. Importantly, its relationship with chest pain – a common manifestation of cardiovascular stress – has not been thoroughly examined. To address this gap, we analyzed data from the 2017 to 2020 National Health and Nutrition Examination Survey (NHANES) to investigate the association between WCS and chest pain. We hypothesized that WCS would be associated with a lower prevalence of chest pain.

## 2. Materials and methods

### 2.1. Study population

Data for this cross-sectional study were obtained from NHANES, a nationally representative survey conducted by the Centers for Disease Control and Prevention (CDC) to monitor health and nutrition among noninstitutionalized Americans. Utilizing a stratified, multistage probability sampling design, NHANES collects extensive health-related information through structured interviews, clinical examinations, and laboratory testing conducted in mobile examination centers. As a publicly available resource, it has long served as a cornerstone for population-based research. The survey protocol received approval from the NCHS Research Ethics Review Board, and all participants provided written informed consent. Further methodological information is available on the official NHANES website (https://www.cdc.gov/nchs/nhanes/).^[14]^

**Figure 1.**
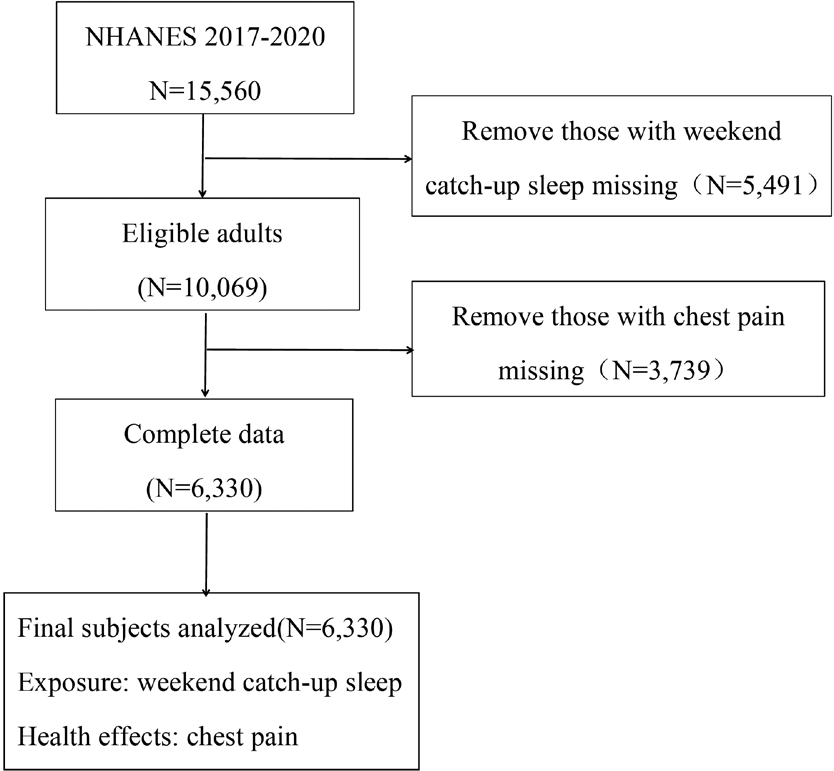
Flow diagram of participant inclusion and exclusion criteria for the analytic sample from NHANES 2017 to 2020. NHANES = National Health and Nutrition Examination Survey, WCS = weekend catch-up sleep.

This analysis was based on NHANES data collected between 2017 and 2020, initially including 15,560 participants. Individuals lacking information on sleep duration (n = 5491) or chest-pain status (n = 3739) were excluded from the study. For other covariates with missing values, we employed multiple imputation to minimize potential bias and to maximize the use of available data. A total of 6330 participants were retained after exclusions for the analysis of WCS in relation to chest pain (Fig. 1). We limited analyses to 2017 to 2020 because the 2021 to 2022 NHANES cycle lacked a harmonized CDQ chest-pain outcome, precluding a same-cycle analysis of WCS and chest pain. The final sample size was sufficiently large to ensure statistical power for the main and subgroup analyses, given the nationally representative design of NHANES.

### 2.2. Chest pain ascertainment

Chest pain status was determined using the NHANES Medical Conditions Questionnaire, which included the question: “Have you ever had any pain or discomfort in your chest?” Participants who answered “yes” were classified as having chest pain, while others were considered chest pain-free. Although this measure relies on self-report, which may introduce misclassification or recall bias, it has been validated in previous epidemiological studies^[2,15]^ and is routinely employed in large-scale population-based research, including NHANES analyses. The Rose Angina Questionnaire, a standardized tool for assessing chest-pain symptoms, was also incorporated into NHANES interviews to enhance diagnostic consistency.

### 2.3. Measurements of WCS

WCS was computed from self-reported sleep duration, defined as weekend hours of sleep minus weekday hours. Sleep duration data were obtained from NHANES sleep-related questionnaire items, specifically: Weekday sleep: “How many hours do you usually sleep on weekdays or workdays?” Weekend sleep: “How many hours do you usually sleep on weekends or non-workdays?”^[16]^ Participants were then stratified into quartiles based on their WCS values: Q1 (<0 hours), Q2 (0 to <1 hour), Q3 (1 to <2 hours), and Q4 (≥2 hours). In all statistical models, Q1 was designated as the reference category. Quartile cutoffs were chosen to capture potential nonlinear associations and are consistent with prior literature examining WCS in relation to health outcomes.^[17]^

### 2.4. Covariates

To control for confounding, we incorporated covariates identified from prior research linking them to both sleep behavior and cardiovascular outcomes. Population characteristics included age (treated as continuous), sex (male or female), and race/ethnicity (Non-Hispanic White, Non-Hispanic Black, Mexican American, Other Hispanic, or Other/Multi-racial). Socioeconomic background was represented by education level (<high school, high school, >high school) and marital status (married/cohabiting, widowed/divorced/separated, or never married). Lifestyle-related factors included smoking status and alcohol consumption. Body mass index (BMI) was assessed and categorized as normal (<25 kg/m²), overweight (25–29.9 kg/m²), or obese (≥30 kg/m²).^[4]^ Clinical conditions were ascertained based on physician-reported diagnoses, medication use, and laboratory evidence. Diabetes was defined by an affirmative response to the question: “Other than during pregnancy, have you ever been told by a doctor or health professional that you have diabetes or sugar diabetes?” Hypertension was identified if participants responded “yes” to: “Have you ever been told by a doctor or other health professional that you had hypertension, also called high blood pressure?” Stroke was confirmed by an affirmative answer to: “Has a doctor or other health professional ever told you that you had a stroke?” A history of heart attack was based on a “yes” response to: “Has a doctor or other health professional ever told you that you had a heart attack, also called myocardial infarction?” These covariates were selected based on established epidemiological evidence linking them to both sleep behaviors and cardiometabolic outcomes.^[18]^ Missing covariate values were imputed using multiple imputation methods, which allowed us to retain participants with partially missing data and reduce the risk of bias due to case-wise deletion. Variables such as sleep quality, depression, and psychosocial stress were not consistently available across NHANES cycles and were therefore not included, which may leave residual confounding.

### 2.5. Statistical analyses

We performed statistical analyses using R (version 4.3.0) alongside Empower Stats (version 5.0). NHANES survey weights were applied to address the complex sampling scheme and yield representative national estimates. Descriptive statistics were presented as weighted means with standard errors for continuous variables and weighted proportions for categorical variables. We evaluated the association between WCS and chest pain using multivariable logistic regression, presenting adjusted odds ratios (ORs) with 95% confidence intervals (CIs). Model 1 adjusted for demographic factors (age, sex, race/ethnicity). Model 2 further included socioeconomic, lifestyle, and clinical variables: education, marital status, alcohol intake, BMI, hypertension, diabetes, history of myocardial infarction, stroke, and smoking. Potential nonlinear relationships were investigated through generalized additive models with spline functions. Significant nonlinear associations (*P* <.05) were further modeled using 2-piecewise linear regression, with breakpoints determined via maximum likelihood. Subgroup analyses considered age, sex, and major cardiovascular risk factors, while interactions were assessed by likelihood ratio testing. A 2-sided *P* <.05 denoted statistical significance.

To test the robustness of the findings, sensitivity analyses excluded individuals in the top and bottom 1% of the WCS distribution. The fully adjusted multivariable logistic regression (Model II) was reestimated in this restricted sample, with adjusted ORs and 95% CIs reported to assess consistency of the association between WCS and chest pain.

## 3. Results

### 3.1. Characteristics of the participant

A total of 6330 participants were included in this study, stratified by WCS quartiles. Table 1 summarizes the baseline characteristics. Participants with greater WCS were generally younger, more likely to be Non-Hispanic Black, and had lower educational attainment. Marital status also differed significantly across quartiles.

**Table 1:** Baseline characteristics of participants across quartiles of WCS in NHANES 2017 to 2020.

Characteristics	Overall	Quartile 1 (WCS < 0)	Quartile 2 (0 ≤ WCS < 1)	Quartile3 (1 ≤ WCS < 2)	Quartile4 (WCS ≥ 2)	*P*-value
N	6330	904	3312	1000	1114	–
Age	60.51 ± 12.04	59.37 ± 11.78	63.80 ± 11.91	56.57 ± 11.10	55.16 ± 10.17	<.001
Sex						
Male	3117 (49.24%)	445 (49.23%)	1676 (50.60%)	474 (47.40%)	522 (46.86%)	.096
Female	3213 (50.76%)	459 (50.77%)	1636 (49.40%)	526 (52.60%)	592 (53.14%)	
Race						
Mexican American	647 (10.22%)	84 (9.29%)	267 (8.06%)	118 (11.80%)	178 (15.98%)	<.001
Other Hispanic	620 (9.79%)	74 (8.19%)	286 (8.64%)	114 (11.40%)	146 (13.11%)	
Non-Hispanic White	2363 (37.33%)	293 (32.41%)	1505 (45.44%)	323 (32.30%)	242 (21.72%)	
Non-Hispanic Black	1686 (26.64%)	326 (36.06%)	699 (21.11%)	278 (27.80%)	383 (34.38%)	
Non-Hispanic Asian	743 (11.74%)	77 (8.52%)	421 (12.71%)	129 (12.90%)	116 (10.41%)	
Other Race – Including Multi-Racial	271 (4.28%)	50 (5.53%)	134 (4.05%)	38 (3.80%)	49 (4.40%)	
Education level						
Less than high school	1341 (21.18%)	187 (20.69%)	708 (21.38%)	183 (18.30%)	263 (23.61%)	.002
High school	1512 (23.89%)	241 (26.66%)	774 (23.37%)	219 (21.90%)	278 (24.96%)	
Above high school	3477 (54.93%)	476 (52.65%)	1830 (55.25%)	598 (59.80%)	573 (51.44%)	
Marital status						
Married/living with partner	3784 (59.78%)	483 (53.43%)	1971 (59.51%)	635 (63.50%)	695 (62.39%)	<.001
Widowed/divorced/separated	1941 (30.66%)	320 (35.40%)	1062 (32.07%)	263 (26.30%)	296 (26.57%)	
Never married	605 (9.56%)	101 (11.17%)	279 (8.42%)	102 (10.20%)	123 (11.04%)	
Diabetes						
Yes	1564 (24.71%)	224 (24.78%)	886 (26.75%)	211 (21.10%)	243 (21.81%)	<.001
No	4766 (75.29%)	680 (75.22%)	2426 (73.25%)	789 (78.90%)	871 (78.19%)	
Heart attack						
Yes	412 (6.51%)	67 (7.41%)	260 (7.85%)	41 (4.10%)	44 (3.95%)	<.001
No	5918 (93.49%)	837 (92.59%)	3052 (92.15%)	959 (95.90%)	1070 (96.05%)	
Stroke						
Yes	453 (7.16%)	93 (10.29%)	268 (8.09%)	39 (3.90%)	53 (4.76%)	<.001
No	5877 (92.84%)	811 (89.71%)	3044 (91.91%)	961 (96.10%)	1061 (95.24%)	
Smoking						
Yes	2846 (44.96%)	452 (50.00%)	1567 (47.31%)	402 (40.20%)	425 (38.15%)	<.001
No	3484 (55.04%)	452 (50.00%)	1745 (52.69%)	598 (59.80%)	689 (61.85%)	
Drinking status						
Yes	4959 (78.34%)	714 (78.98%)	2539 (76.66%)	801 (80.10%)	905 (81.24%)	.004
No	1371 (21.66%)	190 (21.02%)	773 (23.34%)	199 (19.90%)	209 (18.76%)	
BMI						
<25	1285 (20.30%)	164 (18.14%)	713 (21.53%)	200 (20.00%)	208 (18.67%)	.127
25–30	1935 (30.57%)	275 (30.42%)	1013 (30.59%)	315 (31.50%)	332 (29.80%)	
≥30	3110 (49.13%)	465 (51.44%)	1586 (47.89%)	485 (48.50%)	574 (51.53%)	
Hypertension						
Yes	3146 (49.70%)	501 (55.42%)	1723 (52.02%)	439 (43.90%)	483 (43.36%)	<.001
No	3184 (50.30%)	403 (44.58%)	1589 (47.98%)	561 (56.10%)	631 (56.64%)	
Chest pain						
No	4474 (70.68%)	593 (65.60%)	2308 (69.69%)	737 (73.70%)	836 (75.04%)	<.001
Yes	1856 (29.32%)	311 (34.40%)	1004 (30.31%)	263 (26.30%)	278 (24.96%)	

BMI = body mass index, NHANES = National Health and Nutrition Examination Survey, WCS = weekend catch-up sleep.

Clinically, higher WCS was associated with a reduced cardiometabolic burden, including lower prevalence of hypertension, diabetes, heart attack, and stroke. Smoking was more common in lower WCS groups, while alcohol differences were minimal. Notably, the prevalence of chest pain declined significantly across quartiles (*P* <.001), suggesting a potential protective role of WCS.

### 3.2. Association of WCS with chest pain

As presented in Table 2, a clear inverse dose–response association was observed between WCS and chest pain across all models. In the unadjusted analysis, each additional hour of WCS was linked to a lower likelihood of chest pain, and individuals in the highest quartile (≥2 hours) had a substantially reduced risk compared to those with negative WCS. The trend remained significant after adjustment for demographics (Model I) and after further adjustment for socioeconomic, behavioral, and clinical factors (Model II).

**Table 2:** Association between weekend catch-up sleep and chest pain.

Exposure	Non-adjusted model	Model I	Model II
WCS	0.90 (0.86–0.93) <.0001	0.90 (0.87–0.94) <.0001	0.92 (0.88–0.96) <0.0001
Quartile 1 (WCS <0)	Ref	Ref	Ref
Quartile 2 (0 ≤WCS <1)	0.83 (0.71–0.97) 0.0188	0.84 (0.72–0.99) 0.0352	0.91 (0.77–1.07) 0.2599
Quartile 3 (1 ≤WCS <2)	0.68 (0.56–0.83) 0.0001	0.70 (0.58–0.86) 0.0006	0.79 (0.64–0.97) 0.0250
Quartile 4 (WCS ≥2)	0.63 (0.52–0.77) <0.0001	0.65 (0.54–0.79) <0.0001	0.71 (0.58–0.87) 0.0010
*P* for trend	<.0001	<.0001	.0006

Model 1 adjust for: sex, age, race.

Model 2 adjust for: sex, age, race, education level, marital status, drinking status, BMI, hypertension, diabetes, heart attack, stroke, smoking.

WCS = weekend catch-up sleep.

In fully adjusted models, participants in the highest WCS quartile had about 29% lower odds of chest pain compared to the lowest quartile (*P* = .001), with a significant linear trend.

Intermediate quartiles showed a stepwise protective trend, with the association becoming significant in Q3 and strongest in Q4, suggesting that ≥ 2 hours of WCS may be needed for meaningful benefit.

#### 3.2.1. Sensitivity analyses

**Figure 2.**
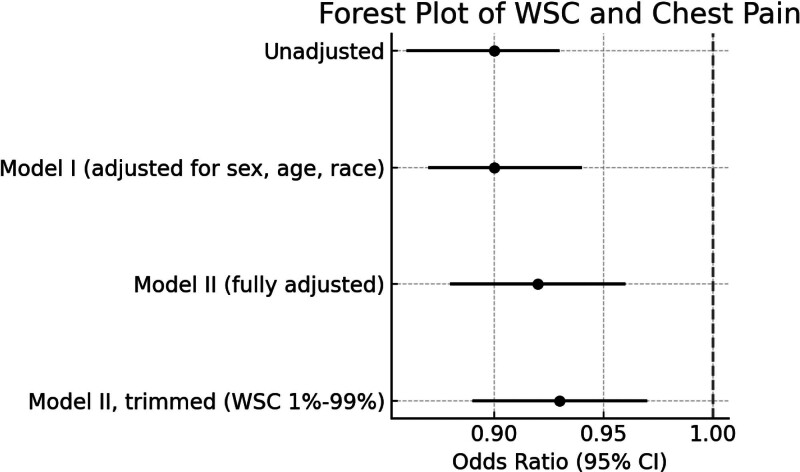
Sensitivity analysis of the association between WCS and chest pain. ORs with 95% CIs are shown from multivariable logistic regression models after excluding participants with extreme WCS values (top and bottom 1%). CI = confidence interval, OR = odds ratio, WCS = weekend catch-up sleep.

To assess the robustness of the association between WCS and chest pain, a sensitivity analysis was performed by excluding participants with extreme WCS values (i.e., the top and bottom 1%). In this trimmed sample, the fully adjusted model (Model II) continued to show a significant inverse association between WCS and chest pain (OR = 0.93, 95% CI: 0.89–0.97; *P* = .0013). This estimate closely mirrored that from the full sample (OR = 0.92, 95% CI: 0.88–0.96), indicating that the observed association was not driven by outliers and remained robust to the influence of extreme values (Table 3 and Fig. 2).

**Table 3:** Sensitivity analysis of the association between WCS and chest pain after excluding participants with extreme WCS values (top and bottom 1%).

Model	OR (95% CI)
Unadjusted	0.90 (0.86–0.93)
Model I (adjusted for sex, age, race)	0.90 (0.87–0.94)
Model II (fully adjusted)	0.92 (0.88–0.96)
Model II, trimmed (WCS 1%–99%)	0.93 (0.89–0.97)

CI = confidence interval, OR = odds ratio, WCS = weekend catch-up sleep.

#### 3.2.2. Restricted cubic spline regression analysis between WCS and chest pain

**Figure 3.**
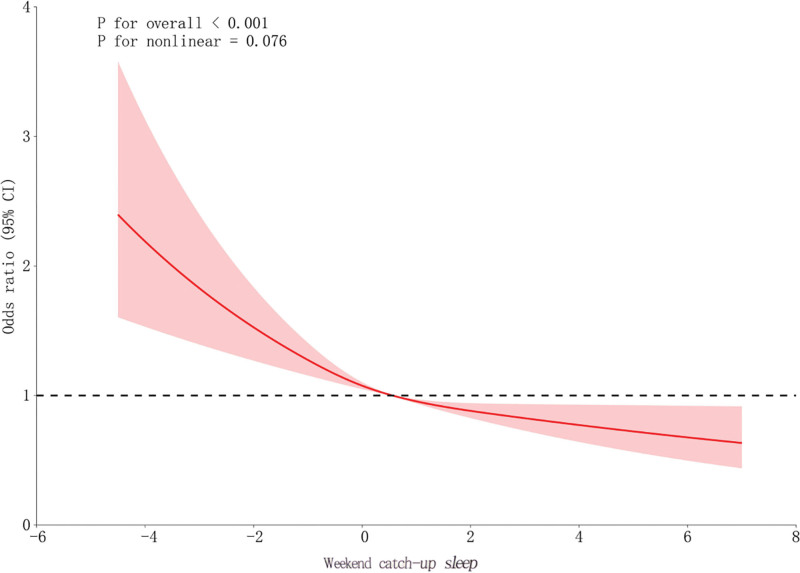
Restricted cubic spline regression showing the dose–response relationship between WCS and chest pain. The solid line represents the estimated ORs, and the shaded area indicates the 95% CIs. CI = confidence interval, OR = odds ratio, WCS = weekend catch-up sleep.

Figure 3 illustrates the nonlinear association between WCS and chest pain. The relationship was statistically significant overall, showing a gradual reduction in chest-pain risk with increasing WCS. As shown in Table 4, a 2-piecewise model suggested an inflection point near–1 hour, though the likelihood ratio test did not confirm a clear threshold. Above–1 hour, each additional hour of WCS was significantly associated with reduced odds of chest pain.

**Table 4:** Two-piecewise logistic regression analysis evaluating the nonlinear relationship between WCS and chest pain.

Chest pain	OR (95% CI), *P*-value
WCS	
Fitting model by standard logistic regression	0.92 (0.88–0.95) <.0001
Fitting model by 2-piecewise logistic regression	–
Inflection point	−1
< −1	0.86 (0.73–1.00) .0562
≥−1	0.93 (0.88–0.97) .0010
*P* for log likely ratio test	.398

Adjusted for all covariates presented in Table 2.

CI = confidence interval, OR = odds ratio, WCS = weekend catch-up sleep.

### 3.3. Subgroup analyses

**Figure 4.**
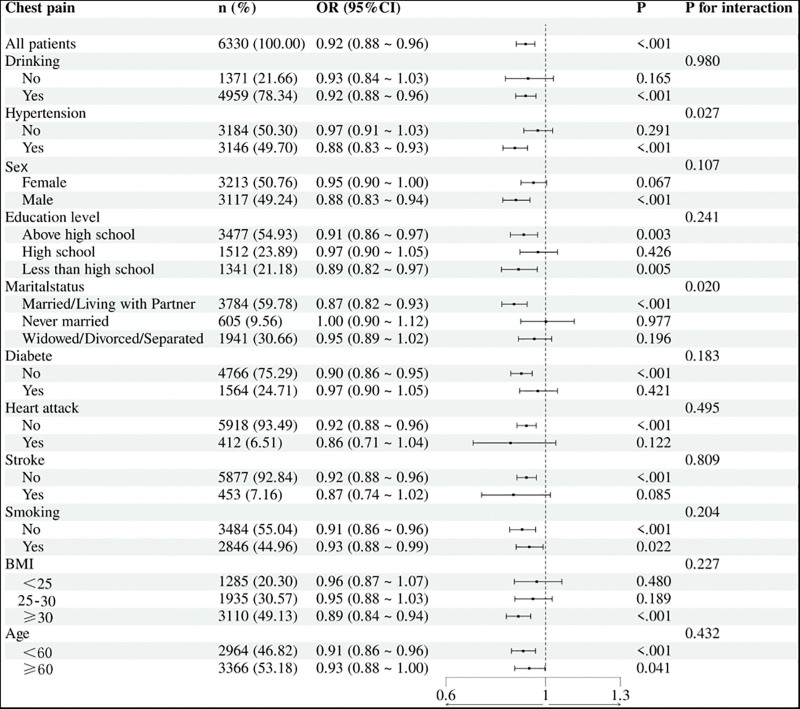
Subgroup analyses of the association between WCS and chest pain. ORs with 95% CIs are displayed for stratified groups (e.g., sex, age, BMI, hypertension, diabetes, marital status). BMI = body mass index, CI = confidence interval, OR = odds ratio, WCS = weekend catch-up sleep.

Figure 4 summarizes subgroup analyses. Significant interactions were observed for hypertension and marital status, with stronger protective effects among hypertensive individuals and those who were married/cohabiting. No significant interactions were found for sex, age, BMI, diabetes, smoking, or prior cardiovascular history, although the overall inverse association remained consistent across most subgroups.

Sex-stratified analysis showed a somewhat stronger effect in males, while obese participants also demonstrated a notable protective association. Overall, WCS was broadly linked to reduced chest-pain risk, particularly in hypertensive and partnered individuals.

## 4. Discussion

Using NHANES 2017 to 2020 data, this study observed an inverse dose–response relationship between WCS and chest pain. Each additional hour of WCS was associated with an 8% lower odds of chest pain (OR = 0.92, 95% CI: 0.88–0.96; *P* <.0001). Sensitivity analyses excluding extreme WCS values (top and bottom 1%) yielded similar results, supporting the robustness of this association.. Participants in the highest WCS quartile (≥2 hours) had a 29% lower risk compared to the lowest quartile (OR = 0.71, 95% CI: 0.58–0.87; *P* <.001). Stronger associations were observed among individuals with hypertension and those who were married or cohabiting. A consistent inverse trend was noted across WCS levels, though the nonlinearity was not statistically significant (*P* = .076). Given the cross-sectional design, these findings should be interpreted as exploratory and hypothesis-generating rather than causal.

Our findings align with emerging evidence linking sleep patterns to cardiovascular health. For instance, Fan et al reported that irregular sleep, including compensatory sleep, may influence cardiovascular risk through autonomic dysfunction and inflammation, consistent with our observed association between WCS and reduced chest-pain prevalence.^[19]^ Similarly, a prospective study by Han, H., et al highlighted the nonlinear relationship between sleep duration and cardiovascular outcomes, which is compatible with our dose–response pattern.^[20]^ However, discrepancies exist with studies like Depner et al, which suggested that weekend recovery sleep fails to mitigate metabolic dysregulation.^[21]^ Differences in study design (cross-sectional vs experimental), populations (nationally representative vs controlled cohorts), and exposure definitions may partly explain these inconsistencies.

The putative protective effect of WCS on chest pain may be mediated through multiple pathways. Experimental studies have shown that sleep deprivation exacerbates sympathetic hyperactivity and endothelial dysfunction, both implicated in chest-pain pathogenesis.^[22,23]^ Animal models further demonstrate that sleep extension reduces oxidative stress and inflammation, key contributors to cardiovascular symptoms.^[24,25]^ Additionally, marital status may modulate this relationship via psychosocial support, which buffers stress-related sleep disturbances and cardiovascular strain.^[8,26]^ Collectively, these mechanistic data are consistent with – but do not prove – our observational findings, suggesting that WCS could be related to lower chest-pain prevalence via autonomic and inflammatory pathways.^[27,28]^

Several limitations of this study should be acknowledged. First, although NHANES provides a large, nationally representative dataset, our analysis was restricted to the 2017 to 2020 cycles because the 2021 to 2022 cycle was not fully available or complete at the time of study design. Second, both sleep duration and chest pain were self-reported, which may introduce recall or misclassification bias. Nevertheless, these measures have been validated and are widely used in epidemiological studies. Third, while we categorized weekend catch-up sleep into quartiles to capture potential nonlinear associations and maintain comparability with prior research, alternative categorizations might yield different results. Fourth, although our sample size was sufficiently large for statistical analyses, residual confounding cannot be excluded. In particular, information on sleep quality, depression, and psychosocial stress was not consistently available across NHANES cycles, which may have influenced the observed associations. Finally, the cross-sectional design of NHANES precludes causal inference, and prospective studies are warranted to confirm these findings.

Given the exploratory nature of this study, future research should prioritize prospective cohort designs with repeated sleep assessments, incorporate objective sleep metrics (e.g., actigraphy, sleep timing/regularity), and more comprehensively measure sleep quality and mental health variables (e.g., depression, perceived stress). Analyses leveraging causal-inference frameworks (e.g., marginal structural models) and randomized sleep-extension interventions could further clarify whether compensatory sleep is linked to reductions in chest pain and through which biological pathways.

## 5. Conclusion

In this cross-sectional analysis of NHANES data, greater WCS was significantly associated with a lower prevalence of chest pain. The inverse association persisted after adjusting for demographic, behavioral, and clinical covariates, and appeared particularly pronounced among individuals with hypertension and those in marital or cohabiting relationships. These findings suggest that compensatory sleep on weekends may play a protective role in mitigating cardiovascular-related symptoms. However, given the cross-sectional nature of the study, causal relationships cannot be inferred. Further prospective and mechanistic studies are warranted to validate these findings and clarify the underlying pathways.

## Acknowledgments

We sincerely thank the dedicated team at the National Center for Health Statistics, part of the Centers for Disease Control and Prevention, for their efforts in designing, collecting, and managing the NHANES data. Their contributions in making this valuable public resource available have greatly advanced public health research.

## Author contributions

**Conceptualization:** Jinjin Jian.

**Data curation:** Jing Zheng.

**Formal analysis:** Jinjin Jian, Qi Wang.

**Investigation:** Jinfang Zeng.

**Software:** Chong Zhao, Jinfang Zeng.

**Writing – original draft:** Chong Zhao, Jing Zheng.

**Writing – review & editing:** Qi Wang.
